# Association of resting heart rate with cardiovascular function: a cross-sectional study in 522 Finnish subjects

**DOI:** 10.1186/1471-2261-13-102

**Published:** 2013-11-15

**Authors:** Jenni K Koskela, Anna Tahvanainen, Antti Haring, Antti J Tikkakoski, Erkki Ilveskoski, Jani Viitala, Miia H Leskinen, Terho Lehtimäki, Mika AP Kähönen, Tiit Kööbi, Onni Niemelä, Jukka T Mustonen, Ilkka H Pörsti

**Affiliations:** 1 School of Medicine, Department of Internal Medicine, University of Tampere, Tampere FIN-33014, Finland; 2 School of Medicine, Department of Pharmacological Sciences, University of Tampere, Tampere, Finland; 3 Heart Center Co., Tampere University Hospital, Tampere, Finland; 4 School of Medicine, Department of Clinical Chemistry, University of Tampere, Tampere, Finland; 5 Department of Clinical Chemistry, Fimlab Laboratories, Tampere, Finland; 6 School of Medicine, Department of Clinical Physiology, University of Tampere, Tampere, Finland; 7 Department of Clinical Physiology, Tampere University Hospital, Tampere, Finland; 8 Laboratory and Medical Research Unit, Seinäjoki Central Hospital, Seinäjoki, Finland; 9 Department of Internal Medicine, Tampere University Hospital, Tampere, Finland

**Keywords:** Arterial stiffness, Cardiac output, Heart rate, Head-up tilt, Systemic vascular resistance

## Abstract

**Background:**

High resting heart rate (HR) is associated with increased cardiovascular risk in general populations, possibly due to elevated blood pressure (BP) or sympathetic over-activity. We studied the association of resting HR with cardiovascular function, and examined whether the hemodynamics remained similar during passive head-up tilt.

**Methods:**

Hemodynamics were recorded using whole-body impedance cardiography and continuous radial pulse wave analysis in 522 subjects (age 20–72 years, 261 males) without medication influencing HR or BP, or diagnosed diabetes, coronary artery, renal, peripheral arterial, or cerebrovascular disease. Correlations were calculated, and results analysed according to resting HR tertiles.

**Results:**

Higher resting HR was associated with elevated systolic and diastolic BP, lower stroke volume but higher cardiac output and work, and lower systemic vascular resistance, both supine and upright (p < 0.05 for all). Subjects with higher HR also showed lower supine and upright aortic pulse pressure and augmentation index, and increased resting pulse wave velocity (p < 0.001). Upright stroke volume decreased less in subjects with highest resting HR (p < 0.05), and cardiac output decreased less in subjects with lowest resting HR (p < 0.009), but clear hemodynamic differences between the tertiles persisted both supine and upright.

**Conclusions:**

Supine and upright hemodynamic profile associated with higher resting HR is characterized by higher cardiac output and lower systemic vascular resistance. Higher resting HR was associated with reduced central wave reflection, in spite of elevated BP and arterial stiffness. The increased cardiac workload, higher BP and arterial stiffness, may explain why higher HR is associated with less favourable prognosis in populations.

**Trial registration:**

ClinicalTrials.gov, NCT01742702

## Background

Measurement of heart rate (HR) is an easily available cardiovascular phenotype in clinical practice. Increased resting HR is associated with higher cardiovascular mortality and morbidity in general populations [1-5], even when other cardiac risk factors are taken into consideration [2,4,5]. Higher HR is also a risk factor for elevated blood pressure (BP) in both children and adolescents [6]. Moreover, many studies have reported that HR is associated with atherosclerosis and elevated risk of adverse cardiovascular events [1,7-9]. However, the results concerning possible benefits of pharmacological HR lowering are inconsistent [7,10-14].

The mechanisms linking elevated HR with cardiovascular pathology and pathophysiology are not well understood. The association has been assumed to be a consequence of sympathetic over-activity [15,16]. In addition, elevated HR and reduced HR variability have been shown to accelerate atherosclerotic process in coronary arteries through local hemodynamic changes [17,18]. Higher resting HR has also been associated with increased pulse wave velocity (PWV), i.e. increased arterial stiffness [19-22]. High PWV is a marker of cardiovascular aging and an acknowledged independent risk factor for cardiovascular morbidity [23].

On the other hand, there is a significant negative association between HR and central BP and central wave reflection [24]. Augmentation index (AIx) is commonly measured to evaluate central wave reflection, and higher AIx can result from increased arterial stiffness, older age, short stature, and lower HR [24]. Increased AIx and higher central wave reflection have also been associated with increased cardiovascular risk [25,26]. Although β-blocking agents seem to increase AIx, this class of drugs appears to have a beneficial influence on prognosis in subjects with coronary artery disease, at least after myocardial infarction [14,24,27]. In addition to the anti-arrhythmic properties of β-blockers, this may be attributed to improved oxygen delivery due to increased time for coronary flow during prolonged diastole [24,27].

Taken together, even though higher HR at rest is associated with lower central wave reflection which is considered to be beneficial, it is associated with less favourable prognosis in observational studies [2,4,5,25,26]. In order to understand the adverse influence of increased HR on prognosis we should identify the underlying hemodynamic differences. Previously, simultaneous analysis of central and peripheral BP, vascular resistance, cardiac function, arterial stiffness and central wave reflection during standard physical challenge has only seldom been performed. The aim of this study was to examine the association of HR with principal hemodynamic variables and their functional responses during head-up tilt in a cross-sectional study including 522 subjects without medications directly influencing HR or BP.

## Methods

### Study population

All study subjects participated in an on-going study, in which hemodynamics are noninvasively recorded from voluntary subjects (DYNAMIC-study; Clinical Trials registration number NCT01742702). The ethics committee of the Tampere University Hospital approved the study protocol and patients gave an informed consent, as stipulated in the Declaration of Helsinki. An announcement for the recruitment of subjects was distributed at the University of Tampere, Tampere University Hospital, several occupational health care organizations, Varala Sports Institute, and two announcements were published in a local newspaper. The subjects who responded were successively recruited in the order that they contacted the research nurse. In November 2012 a total number of 830 subjects had been recruited to the study.

In present investigation, those subjects with a history of coronary artery disease, diabetes mellitus, peripheral arterial or cerebrovascular disease, valvular regurgitation or stenosis, long QT syndrome, chronic renal insufficiency, hemochromatosis, or medication for hypertension were excluded. Also, subjects with regular medication influencing HR or BP were excluded, i.e. subjects using anti-arrhythmic agents, long-acting β_2_-sympathomimetics, α-adrenoceptor agonists, varenicline, or the weight-reducing agent sibutramine.

Altogether 522 subjects (261 males, aged 20 to 72 years) with technically successful hemodynamic recordings were included in the present study. The majority of subjects were without concurrent diseases or medications (for medication details please see Additional file 1: Table S1). In total 80 of 261 women were on low-dose progesterone (intrauterine device) or combination of oestrogen and progesterone therapy (contraception or hormone replacement therapy). Subjects with the following medical conditions with established and stable drug treatment were included in the study: depression (n = 29), allergies or asthma (n = 26), dyspepsia (n = 15), hypothyroidism (n = 15), hyperlipidaemia (n = 14), musculoskeletal problems (n = 10), and epilepsy (n = 5). All subjects with thyroid problems were euthyroid clinically and on the basis of laboratory tests. Moreover, mean HR was not statistically different between any medicated versus corresponding unmedicated subgroups of subjects.

All subjects underwent a physical examination performed by a physician, who also documented medical history, lifestyle habits, and cardiovascular risk factors by interview. The amount of smoking was calculated in pack years, and the use of alcohol was evaluated as average consumption of standard drinks (corresponding to 12 grams of absolute alcohol) per week. Physical exercise frequency was interviewed, and was expressed as the number of bouts of physical activity per week that lasted for at least 30 min each time and caused sweating or shortness of breath.

### Laboratory analyses

Venous blood samples were drawn after ~12 hours of fasting. Plasma sodium, potassium, glucose, creatinine, cystatin C, C-reactive protein (CRP), and total, high-density (HDL) and low-density lipoprotein (LDL) cholesterol concentrations were determined using Cobas Integra 700/800 (F. Hoffmann-LaRoche Ltd, Basel, Switzerland), or Cobas 6000, module c501 (Roche Diagnostics, Basel, Switzerland), and white blood cell count and haematocrit using ADVIA 120 or 2120 analyzers (Bayer Health Care, Tarrytown, NY, USA). Glomerular filtration rate was estimated using the Rule formula [28], since the measured creatinine values were within the normal range.

### Hemodynamic measurements

Hemodynamics recordings were carried out in a quiet, temperature-controlled laboratory by a research nurse. The subjects were instructed to refrain from caffeine-containing products, smoking, and heavy meals for at least 4 h, and from alcohol for at least 24 h prior to the investigation. Before the actual measurement the subjects were resting supine for approximately 10 min, during which period electrodes for impedance cardiography were placed on the body surface, a tonometric sensor for pulse wave analysis was fixed to the left wrist on the radial pulsation, and a brachial cuff for BP calibration was placed to the right upper arm. Then hemodynamic variables were continuously captured in a beat-to-beat fashion for 5 min in supine position and for 5 min during passive head-up tilt to 60 degrees. Mean values of each measured minute of the experiment were calculated and used in statistical analyses.

### Whole-body impedance cardiography

A whole-body impedance cardiography device (CircMon^R^, JR Medical Ltd, Tallinn, Estonia), which records the changes in body electrical impedance during cardiac cycles, was used to determine beat-to-beat HR, stroke index (stroke volume in proportion to body surface area, ml/m^2^), cardiac index (cardiac output/body surface area, l/min/m^2^), and PWV (m/s) [29-31]. Left cardiac work index (kg*m/min/m^2^) was calculated by formula 0.0143*(MAP–PAOP)*cardiac index, which has been derived from the equation published by Gorlin et al. [32]. MAP is mean radial arterial pressure measured by tonometric sensor, PAOP is pulmonary artery occlusion pressure which is assumed to be normal (default 6 mmHg), and 0.0143 is the factor for the conversion of pressure from mmHg to cmH_2_O, volume to density of blood (kg/L), and centimetre to metre. Systemic vascular resistance index (systemic vascular resistance/body surface area, dyn*s/cm^5^/m^2^) was calculated from the signal of the tonometric BP sensor and cardiac index measured by CircMon^R^.

To calculate the PWV, the CircMon software measures the time difference between the onset of the decrease in impedance in the whole-body impedance signal and the popliteal artery signal. From the time difference and the distance between the electrodes, PWV can be determined. As the whole-body impedance cardiography slightly overestimates PWV when compared with Doppler ultrasound method, a validated equation was utilized to calculate values that correspond to the ultrasound method (PWV = (PWV_impedance_*0.696) + 0.864) [30]. PWV was determined only in the supine position because of less accurate timing of left ventricular ejection during head-up tilt [30]. A detailed description of the method and electrode configuration has been previously reported [31]. PWV was also recorded after the head-up tilt in all subjects, and the average difference between the mean PWV before and after the head-up tilt was 0.024 ± 0.388 m/s (mean ± standard deviation), showing the good repeatability of the method (repeatability index R 98%, Bland-Altman repeatability index 0.8) [33]. The cardiac output values measured with CircMon^R^ are in good agreement with the values measured by the thermodilution method [31], and the repeatability and reproducibility of the measurements (including PWV recordings) have been shown to be good [34,35].

### Pulse wave analysis

Radial BP and pulse wave form were continuously determined by the use of an automatic tonometric sensor (Colin BP-508 T, Colin Medical Instruments Corp., USA), which was fixed on the radial pulse with a wrist band. The extended left arm was lying on a stable bracket at the level of the heart, whether supine or upright. Radial BP signal was calibrated by brachial BP measurement at the onset of the recording. Continuous aortic BP was derived with the SphygmoCor monitoring system (SphygmoCor PWMx, AtCor Medical, Australia) using the previously validated generalized transfer function [36]. From the aortic pulse wave form AIx (augmented pressure/pulse pressure*100,%) was determined.

### Stroke volume determination with cardiac ultrasound

To evaluate the accuracy of stroke volume determination with impedance cardiography during head-up tilt, echocardiography was performed by a cardiologist (author E.I.) to a subset of subjects (n = 16) during an extra visit. The 3D echocardiography (Philips ie33 ultrasound system, Bothell, USA; 1-5 MHz Matrix-array X5-1 transducer) was performed simultaneously with beat-to-beat impedance cardiography recordings during head-up tilt to 60 degrees. Mean stroke volume from 7 consecutive heart beats (6 before and 1 after echocardiography) was calculated from impedance cardiography recordings to cover approximately one respiratory cycle (~6 seconds).

### Statistical analyses

For the statistical analyses, the study population was divided into tertiles according to mean resting HR, determined as an average HR of the last 3 min during the 5-min measurement period in supine position. Analysis of variances for repeated measures was applied to study the differences in the hemodynamic variables BP, stroke index, cardiac index, left cardiac work index, systemic vascular resistance index, AIx, and PWV between the HR tertile groups during rest and head-up tilt. For post hoc testing Tukey HSD test was performed for homogenous, and Tamhane’s T2 test for nonhomogeneous variables. In adjusted analysis of variances for repeated measures, the variables sex, age, body mass index, smoking in pack years, haematocrit, leukocyte count, CRP, creatinine, cystatin C, total cholesterol, triglycerides, HDL cholesterol, fasting plasma glucose, and mean radial arterial pressure at rest were used as covariates.

Pearson’s correlation coefficients were calculated, as appropriate, and possible differences in stroke volume determined using impedance cardiography and 3D echocardiography were tested using Student’s T-test. Distributions of categorical variables among resting HR tertiles were tested using χ^2^ test, and differences of numerical variables among HR tertiles were studied using analysis of variances. Variable values are given as means and 95% confidence intervals (CI). Natural logarithms of CRP and triglyceride concentrations were used in analyses to normalize their distributions. P-values <0.05 were considered statistically significant. The analyses were performed using SPSS Statistics 17.0 for Windows software (SPSS Inc., Chicago, Ill., USA).

## Results

### Study population

The characteristics of the study population according to the HR tertiles at rest, with average values of 54, 62 and 75 beats/min in the 1st, 2nd and 3rd tertile, respectively, are shown in Table 1. Mean resting HR among men was 62 (CI: 61 to 64), and among women 64 (CI: 63 to 65) beats/min (p = 0.028). The proportion of men was higher in tertile 1 (with lowest HR) when compared with tertiles 2 and 3 (p = 0.019). Age, use of alcohol, amount of smoking, hematocrit, and plasma concentrations of sodium, potassium, HDL cholesterol, LDL cholesterol, and glucose did not differ between the HR tertiles (p > 0.05 for all). Body mass index, white blood cell count, C-reactive protein, plasma total cholesterol and triglycerides were highest within tertile 3 (p < 0.05 for all). Although plasma creatinine was lowest in tertile 3 (p < 0.005), there were no significant differences in cystatin-C concentrations or estimated glomerular filtration rate between the groups. The self-reported amount of physical exercise bouts per week was 3.4 in tertile 1 (CI: 3.1 to 3.7), 3.0 in tertile 2 (CI: 2.7 to 3.3), and 3.1 in tertile 3(CI: 2.7 to 3.5), and the differences between the tertiles were not statistically significant (p = 0.143).

**Table 1:** Characteristics of the study population

	**Resting heart rate tertiles**		
	**1**	**2**	**3**
	**n = 172**	**n = 176**	**n = 174**
Resting heart rate (1/min)	54 (53–54)	62 (62–63)*	75 (73–75)*^†^
Age (years)	46 (44–48)	46 (44–47)	46 (44–47)
Sex (M/F)	101/71^‡^	79/97	81/93
Body mass index (kg/m^2^)	26.2 (25.7–26.7)	26.5 (25.8–27.1)	27.3 (26.6–28.1)*
Waist circumference (cm)	92 (90–93)	92 (90–94)	94 (92–96)
Smoking (pack years)	2.3 (1.2–3.4)	1.8 (0.9–2.6)	3.5 (2.0–5.0)
Alcohol (drinks/week)	4 (3–5)	4 (3–5)	5 (4–6)
Leukocyte count (1*10^9^/l)	5.4 (5.2–5.6)	5.9 (5.6–6.1)*	6.1 (5.8–6.4)*
Haematocrit (%)	42 (42–43)	41 (41–42)	42 (41–43)
C-reactive protein (mg/l)	1.2 (1.0–1.4)	1.6 (1.2–2.0)	2.2 (1.6–2.9)*
Creatinine (μmol/l)	77 (75–79)	72 (70–74)*	71 (70–73)*
Estimated GFR (ml/min/1.73 m^2^)	112 (109–114)	111 (110–113)	112 (110–114)
Cystatin C (mg/l)	0.83 (0.81–0.85)	0.82 (0.80–0.84)	0.86 (0.83–0.88)
Sodium (mmol/l)	140 (140–141)	140 (140–141)	140 (140–140)
Potassium (mmol/l)	3.8 (3.8–3.9)	3.8 (3.8–3.9)	3.8 (3.7–3.8)
Fasting plasma			
Total cholesterol (mmol/l)	5.2 (5.0–5.4)	5.0 (4.8–5.1)*	5.2 (5.1–5.4)^†^
Triglycerides (mmol/l)	1.1 (1.0–1.2)	1.2 (1.1–1.3)	1.3 (1.2–1.5)*^†^
HDL cholesterol (mmol/l)	1.7 (1.6–1.7)	1.6 (1.5–1.6)	1.5 (1.5–1.6)
LDL cholesterol (mmol/l)	3.0 (2.9–3.2)	2.9 (2.7–3.0)	3.1 (3.0–3.3)
Glucose (mmol/l)	5.4 (5.3–5.5)	5.4 (5.3–5.4)	5.5 (5.4–5.6)

Values are means and 95% confidence intervals; *p < 0.05 vs. tertile 1, ^†^p < 0.05 vs. tertile 2, analysis of variance with Tukey HSD post hoc test; ^‡^p < 0.05 for distributions between the tertiles, χ^2^ test; GFR = glomerular filtration rate using the Rule formula [28].

### BP in the HR tertiles

**Figure 1:**
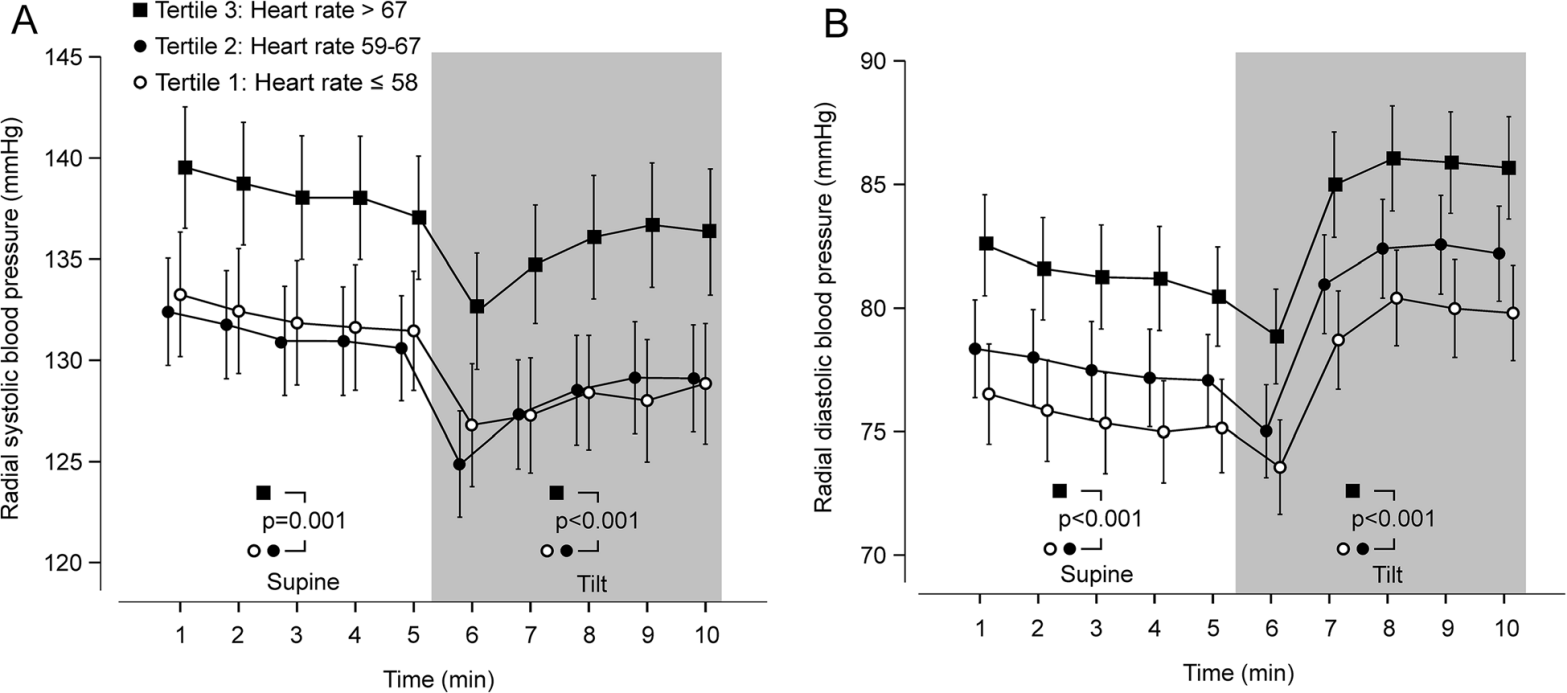
**Radial blood pressures during rest and passive head-up tilt according to resting heart rate tertiles. (A)** Systolic blood pressure, **(B)** diastolic blood pressure; mean (95% confidence intervals), p values denote significant differences between the tertiles depicted with different symbols.

In the entire study population HR at rest showed a moderate association with resting radial systolic BP (r = 0.14, p < 0.001) and diastolic BP (r = 0.19, p < 0.001). In tertile 3 radial systolic and diastolic BPs were higher than in other tertiles, in both supine position and during head-up tilt (Figure 1). The differences in radial BPs between HR tertile 3 versus other tertiles in supine and upright positions remained significant in adjusted analyses including the covariates given in the Statistical analyses section of Methods.

Aortic systolic BP did not differ between the tertiles in supine (p = 0.139) or upright positions (p = 0.452). However, supine aortic diastolic BP was higher in tertile 3 than tertiles 1 and 2 (p ≤ 0.033, unadjusted and adjusted comparisons). Upright aortic diastolic BP was higher in tertile 3 than tertile 1 (p < 0.001).

### Stroke volume, cardiac work, and systemic vascular resistance among HR tertiles

**Figure 2:**
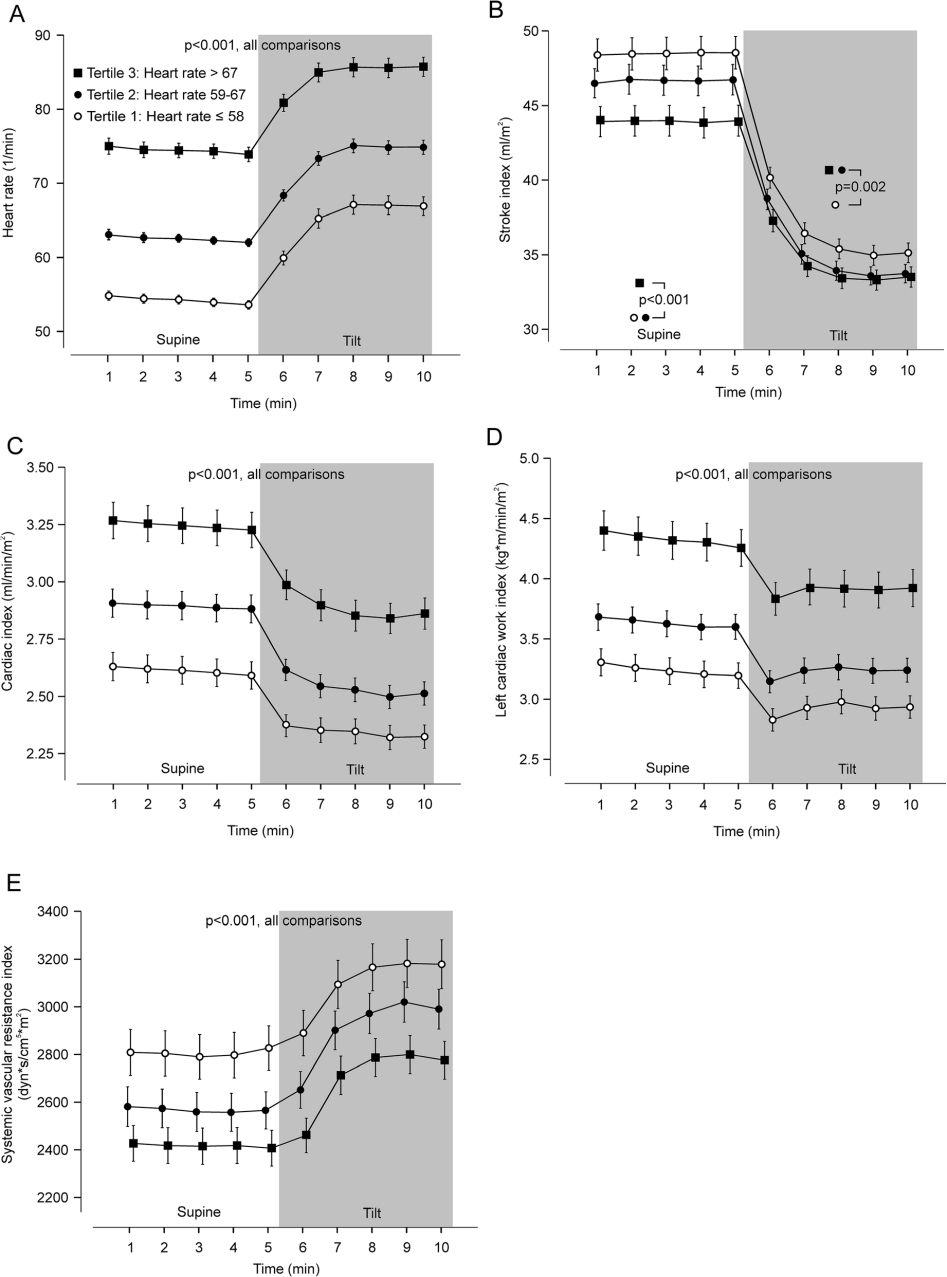
**Principal hemodynamic variables during rest and passive head-up tilt according to resting heart rate tertiles. (A)** Heart rate, **(B)** stroke index, **(C)** cardiac index, **(D)** left cardiac work index, **(E)** systemic vascular resistance index; mean (95% confidence intervals).

All HR tertiles showed a corresponding upright chronotropic effect (11.5-13.1 beats/min, p > 0.05), and the clear differences in HR between the tertiles persisted during the head-up tilt (Figure 2A). The mean supine to upright increase in HR in the whole population was 12.4 (CI: 11.8 to 12.9) beats/min.

Supine stroke index showed a significant negative correlation with HR (r = -0.31, p < 0.001), and in tertile 3 supine stroke index was lower than in other tertiles (Figure 2B, p < 0.001 also after adjustments). In response to head-up tilt, the decrease in stroke index was smaller in the 3rd tertile (10.4 ml/m^2^, CI: 9.7 to 11.2) , than in the 1st (13.3 ml/m^2^, CI: 12.4 to 14.2), and 2nd tertiles (12.9 ml/m^2^, CI: 12.0 to 13.7) (unadjusted and adjusted p < 0.001). Upright stroke index was lower in tertiles 2 and 3 than in tertile 1 (p = 0.002).

In spite of the above negative correlation between stroke index and HR, supine cardiac index and also left cardiac work index were highest in tertile 3, and were also higher in tertile 2 than tertile 1 (Figures 2C and 2D, p < 0.001 also in adjusted analyses). The upright decrease in cardiac index was slightly higher in the 2nd (-0.38 ml/min/m^2^, CI: -0.31 to -0.44), and 3rd tertiles (-0.38 ml/min/m^2^, CI: -0.32 to -0.44), than in the 1st tertile (-0.28 ml/min/m^2^, CI: -0.22 to -0.33) (p < 0.034), but in adjusted analyses the difference remained significant only between the 1st and 3rd tertiles (p < 0.009). Importantly, the clear differences in cardiac index and left cardiac work index between the HR tertiles persisted in the upright position (p < 0.001 in unadjusted and adjusted analyses).

Systemic vascular resistance index was lowest in tertile 3, and also lower in tertile 2 than tertile 1, and the differences remained significant in the upright position (Figure 2E, p < 0.001 also after adjustments).

### HR, central wave reflection, and arterial stiffness

**Figure 3:**
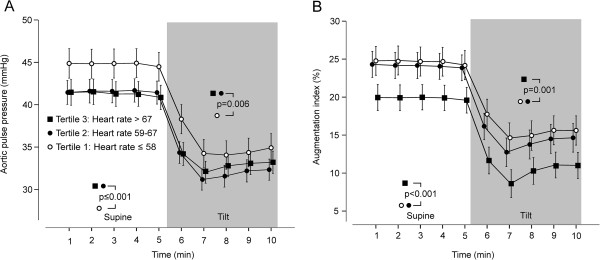
**Central pulse pressure and wave reflection during rest and passive head-up tilt according to resting heart rate tertiles. (A)** Aortic pulse pressure, **(B)** augmentation index; mean (95% confidence intervals).

In the whole population, an expected negative association was found between resting HR and AIx (r = -0.19, p < 0.001), so that AIx was mathematically reduced by 2.5%-units for every 10 beats/min increase in HR. Supine aortic pulse pressure was highest in the 1st HR tertile (Figure 3A), while supine AIx was lowest in the 3rd HR tertile (Figure 3B), and corresponding differences were also observed during the head-up tilt. The differences in aortic pulse pressure and AIx remained significant in adjusted analyses (p ≤ 0.001 for all, supine and upright). As the subject’s height may influence AIx, we performed an additional analysis so that body mass index was replaced by height and weight in the adjustments, but the outcome of the analysis did not change.

**Figure 4:**
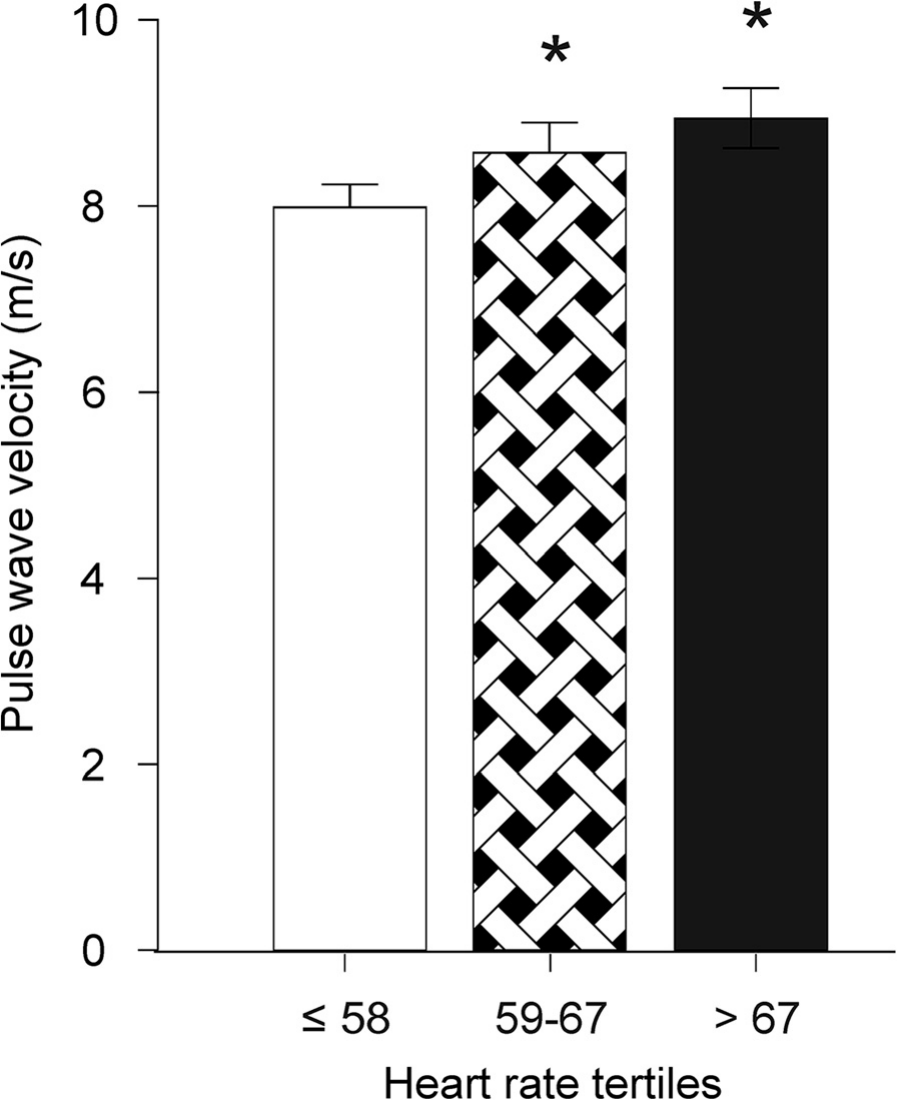
Pulse wave velocity according to resting heart rate tertiles; mean (95% confidence intervals), *p < 0.001 vs. tertile 1.

Arterial stiffness was evaluated by measuring resting PWV, the mean value of which in the whole study population was 8.5 m/s (CI: 8.3 to 8.7). HR was notably correlated with PWV (r = 0.23, p < 0.001). PWV was significantly higher in tertiles 2 and 3 than in tertile 1 (Figure 4; p < 0.001 also in adjusted analysis).

### Determination of stroke volume using impedance cardiography and echocardiography

**Figure 5:**
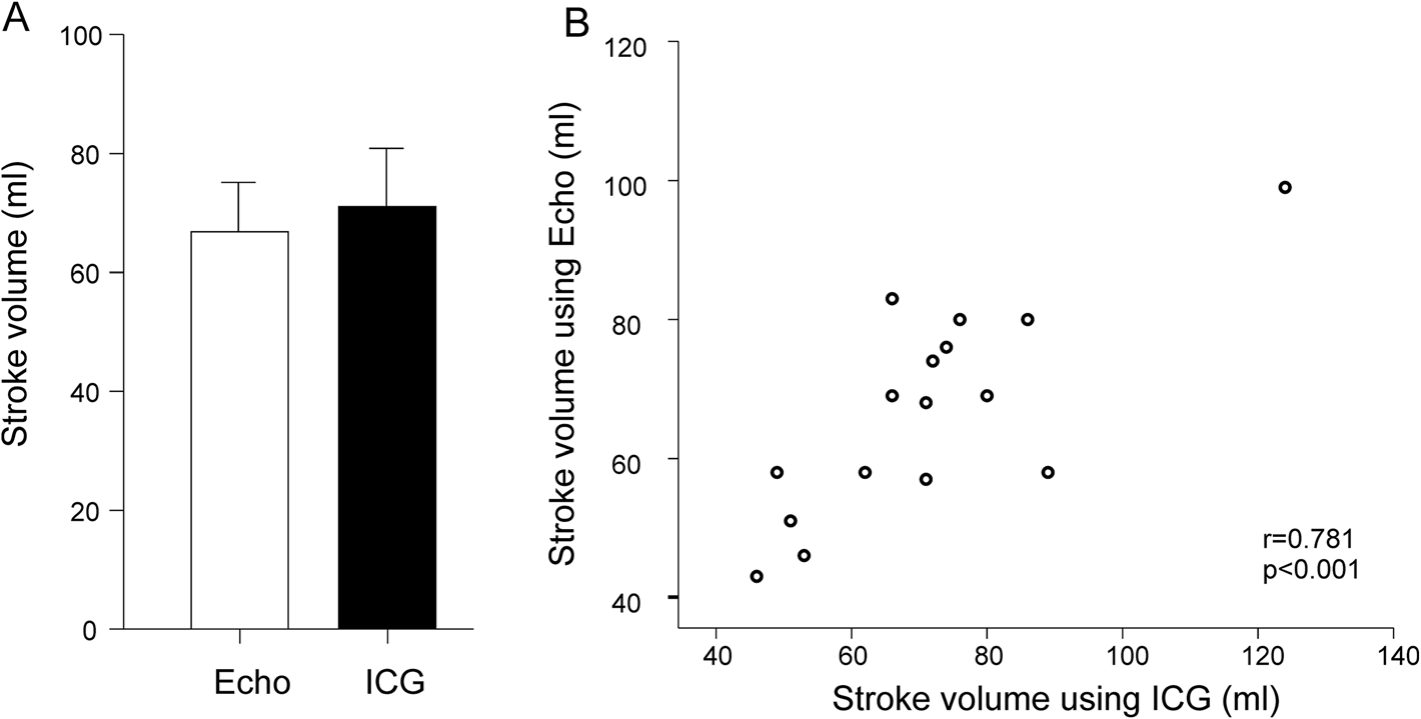
**Evaluation of stroke volume in 16 subjects during passive head-up tilt using echocardiography (Echo) and impedance cardiography (ICG). (A)** Comparison of stroke volume measurements mean (95% confidence intervals). **(B)** Scatterplot depicting the measurements in each individual.

Stroke volume during the head-up tilt was determined by means of impedance cardiography and cardiac 3D ultrasound in 16 subjects. Mean stroke volume by impedance cardiography was 72 ml (CI: 61 to 82) and by echocardiography 67 ml (CI: 59 to 75) (p > 0.05, Figure 5A). The correlation between the impedance cardiography and echocardiography recordings was good (Figure 5B).

## Discussion

To our knowledge, the association of resting HR with cardiovascular response to head-up tilt has not been studied previously. Here we demonstrated that higher resting HR was associated with reduced central wave reflection and lower vascular resistance, but in spite of these beneficial characteristics, cardiac output and work were increased in tertiles with higher resting HR, both supine and upright. Moreover, BP was moderately increased in the 3rd HR tertile, while arterial stiffness was increased in both 2nd and 3rd HR tertiles when compared with the lowest HR tertile. Altogether, the present findings support the view that lower resting HR represents a more beneficial hemodynamic profile.

Since the change in posture induces significant hemodynamic changes in blood volume distribution, vascular resistance, and autonomic nervous function, head-up tilt can be regarded as a test addressing cardiovascular reactivity [37,38]. The head-up tilt -induced decrease in stroke volume was slightly lesser in subjects with highest resting HR, while the decrease in cardiac output was somewhat lower in the subjects with lowest resting HR, despite the changes in HR between the tertiles did not differ. However, the clear differences in cardiac output and vascular resistance between the HR tertiles persisted in the upright position, and the observed differences between the hemodynamic profiles in the HR tertiles were surprisingly similar both supine and upright. This suggests that resting HR provides significant information about upright hemodynamics, so that higher resting HR indicates a more hyperdynamic upright hemodynamic profile.

The increased cardiovascular risk related to higher resting HR has been attributed to genetic factors, impaired myocardial oxygen delivery, increased BP, arrhythmias, sympathetic over-activity, and increased arterial stiffness [1-5,7,15,16], but the mechanisms are not completely understood. Elevated BP is considered one of the most potential mechanisms associating higher HR with cardiovascular risk [6,39]. In the present study, despite lower stroke volume and vascular resistance, radial BP was highest in the 3rd tertile with the highest resting HR, in line with previous results [39]. However, as the difference in central and peripheral BP between the HR tertiles was rather small, other factors in addition to elevated BP may play a more important pathophysiological role in the cardiovascular risk associated with elevated HR. Moreover, increased HR may merely be a marker, but not a cause, for higher risk of cardiovascular end-points.

A change in heart rate is a major factor by which the cardiovascular system adjusts cardiac output [40], and in the absence of a heart disease, higher HR is commonly assumed to indicate higher cardiac output. Although cardiac output cannot be reliably predicted from HR alone [40], the present results suggest that higher HR is associated with increased cardiac output. The known determinants of cardiac output are the interaction of i) cardiac function, which is determined by heart rate, contractility, afterload and preload; and ii) return function, which is determined by vascular volume, venous compliance, blood draining from the venous compliant regions, and right atrial pressure (for a review, see [40]).

We found that higher HR was related with increased left cardiac work, and thus higher cardiac oxygen demand. In patients with coronary artery disease, reducing HR is an acknowledged treatment modality, which reduces myocardial oxygen consumption and improves subendocardial blood flow [14,24,27]. In an experimental study in dogs, increasing HR was found to increase cardiac oxygen demand even when the external work performed by the heart was kept constant, and this effect was attributed to the greater oxygen requirement for excitation-contraction coupling during higher HR [41]. Moreover, although the benefits of HR lowering in the treatment of hypertension have recently been questioned [10-13], pharmacological reduction of HR and the subsequent decrease in cardiac workload by the use of drugs like β-adrenoceptor blockers might still benefit distinct subgroups of patients [15,16].

Higher resting HR is thought to reflect enhanced sympathetic tone, and this may predispose to cardiac arrhythmias and hypertension [6,42,43]. In the heart increased sympathetic tone has both chronotropic and inotropic effects, while in the resistance arteries higher sympathetic tone induces vasoconstriction and thus elevates BP [44]. Counteracting these effects, parasympathetic tone plays an important role in the regulation of HR and cardiac output, and it also influences vascular resistance via secondary mechanisms [45]. In this study, we observed an inverse relation between higher HR and systemic vascular resistance and stroke index, and these findings seem to contradict with the concept of sympathetic over-activity as the cause of higher HR. On the other hand, higher HR was associated with higher BP, which suggests that vascular resistance was not sufficiently reduced to compensate for the increased cardiac output resulting from higher HR. Therefore, lower resistance in peripheral arteries cannot exclude the possibility of increased sympathetic tone in subjects with higher HR. A thorough analysis of autonomic tone would require the recording of HR variability, baroreceptor sensitivity, or direct muscle sympathetic nerve activity, and such analyses make an interesting topic for further investigations.

We found an inverse relation with HR and AIx and central pulse pressure, in line with previous reports [13,24,46]. Increased AIx, which is an indicator of central wave reflection, has been related to elevated cardiovascular risk [25,26]. When higher HR leads to shorter duration of systole, this shifts the reflected wave towards diastole, and the reduction in AIx during higher HR can thus be regarded as a beneficial hemodynamic change [24]. The present results suggest that the relationship between HR and AIx could also arise from the inverse association of HR with systemic vascular resistance: the reflection point of the forward arterial pressure wave is shifted more peripherally during lower systemic vascular resistance index, and this prolongs the time to wave reflection shifting it towards diastole [24].

Our results showed a small but significant relationship between higher HR and increased PWV, a marker of arterial stiffness [23], in agreement with previous studies [19,22,47,48]. However, HR itself may be an important confounder during PWV assessment. Higher HR exerted a significant increasing influence on PWV in 22 elderly subjects during cardiac pacing, in the absence of changes in BP [19]. In a study with 102 young, healthy males, left ventricular ejection time was an important determinant of PWV both under resting conditions and during adrenergic stimulation: shorter ventricular ejection time was associated with higher PWV [49]. Therefore, the associations between HR and PWV must be interpreted with caution. Although higher arterial stiffness increases pulse pressure [50], we found that aortic pulse pressure was lower in the highest HR tertile when compared with the lowest HR tertile, in spite of higher PWV in the former group. This can be explained by the lower augmentation index during higher heart rate, i.e. lower summation of the reflected pressure wave to the systolic volume wave. In addition, pulse pressure is significantly determined by stroke volume [50], and stroke index was inversely correlated (r = -0.31) with heart rate. Thus, lower aortic pulse pressure in the highest versus lowest heart rate tertile was probably a consequence of reduced augmentation index and lower stroke volume in the former group.

The majority of the present subjects were devoid of medications or prevalent diseases, while those with a diagnosed disorder were in a stable condition and on a constant medication without direct cardiovascular influences. HR was slightly higher in women versus men (64 vs. 62 beats/min), while there was no relation between HR and age. Previously, resting HR has been reported to be 2–7 beats/min higher in female than male subjects, while the effect of age on resting HR has been minor [1,39,51]. Importantly, age and sex were included as confounding variables in the statistical analyses. On the basis of a recent epidemiological FINRISKI survey, the present study population with a mean body mass index of 26.7, total cholesterol level of 5.1 mmol/l, and 18% proportion of smokers, represented well the prevalent Finnish population [52]. The applied methods were non-invasive, safe, and easy to perform, and the accuracy of pulse wave analysis and impedance cardiography has been tested against invasive methods [30,31,53]. To strengthen the results, we performed a small validation study that showed a good correlation between impedance cardiography and echocardiography -derived stroke volume.

When measuring the human hemodynamics noninvasively, many of the variables are calculations or derivatives, and most of the calculations include HR in the procedure. The formulas used here have been found to be reliable [30-32,54], but HR is actually a major determinant in most of the calculated cardiac and vascular hemodynamic variables. Furthermore, some of the background characteristics were strongly correlated with each other, like the associations of body mass index with age, sex, and fasting glucose. These points cause a multicollinearity problem, which cannot be completely controlled for by statistical methods, but has to be taken into consideration when evaluating the results of this study.

## Conclusions

We found that the hemodynamic profile associated with higher resting HR was characterized by higher cardiac output, higher cardiac workload, and lower systemic vascular resistance. The hemodynamic features of the distinct HR tertile groups persisted during the head-up tilt. Higher resting HR was also associated with lower augmentation index and aortic pulse pressure, in spite of elevated BP and arterial stiffness. Thus, possible factors associating higher resting HR with less favourable prognosis in the population studies are higher cardiac workload, elevated BP, and increased arterial stiffness.

## Abbreviations

AIx: Augmentation index; BP: Blood pressure; CI: Confidence interval; CRP: C-reactive protein; HDL: High-density lipoprotein; HR: Heart rate; LDL: Low-density lipoprotein; PWV: Pulse wave velocity.

## Competing interests

The authors have no conflicts of interest to disclose.

## Authors’ contributions

JK, AT, IP, JM designed and conducted the study. JK, AT, IP, AJT analysed and interpreted the data, and drafted the first version of the manuscript. JK, AT, AJT, AH, ML, IP, EI, ON and JV performed experiments. JM, AJT, EI, ON, TL, TK and MK gave critical intellectual input and contributed to drafting revised versions of the manuscript. All authors read and approved the final manuscript.

## Pre-publication history

The pre-publication history for this paper can be accessed here:

http://www.biomedcentral.com/1471-2261/13/102/prepub

## Supplementary Material

Additional file 1 Regular medications used by the study population. Click here for file

## References

[B1] Kannel WB Kannel C Paffenbarger RS Jr Cupples LA Heart rate and cardiovascular mortality: the Framingham Study Am Heart J 1987 113 6 1489 1494 10.1016/0002-8703(87)90666-1 3591616

[B2] Palatini P Benetos A Grassi G Julius S Kjeldsen SE Mancia G Narkiewicz K Parati G Pessina AC Ruilope LM Identification and management of the hypertensive patient with elevated heart rate: statement of a European Society of Hypertension Consensus Meeting J Hypertens 2006 24 4 603 610 10.1097/01.hjh.0000217838.49842.1e 16531784

[B3] Reunanen A Karjalainen J Ristola P Heliövaara M Knekt P Aromaa A Heart rate and mortality J Intern Med 2000 247 2 231 239 10.1046/j.1365-2796.2000.00602.x 10692086

[B4] Jouven X Empana JP Schwartz PJ Desnos M Courbon D Ducimetiere P Heart-rate profile during exercise as a predictor of sudden death N Engl J Med 2005 352 19 1951 1958 10.1056/NEJMoa043012 15888695

[B5] Cooney MT Vartiainen E Laatikainen T Juolevi A Dudina A Graham IM Elevated resting heart rate is an independent risk factor for cardiovascular disease in healthy men and women Am Heart J 2010 159 4 612 619 e613 10.1016/j.ahj.2009.12.029 20362720

[B6] Fernandes RA Freitas Junior IF Codogno JS Christofaro DG Monteiro HL Roberto Lopes DM Resting heart rate is associated with blood pressure in male children and adolescents J Pediatr 2011 158 4 634 637 10.1016/j.jpeds.2010.10.007 21095617

[B7] Julius S Palatini P Kjeldsen SE Zanchetti A Weber MA McInnes GT Brunner HR Mancia G Schork MA Hua TA Usefulness of heart rate to predict cardiac events in treated patients with high-risk systemic hypertension Am J Cardiol 2012 109 5 685 692 10.1016/j.amjcard.2011.10.025 22169130

[B8] Gillman MW Kannel WB Belanger A D’Agostino RB Influence of heart rate on mortality among persons with hypertension: the Framingham Study Am Heart J 1993 125 4 1148 1154 10.1016/0002-8703(93)90128-V 8465742

[B9] Diaz A Bourassa MG Guertin MC Tardif JC Long-term prognostic value of resting heart rate in patients with suspected or proven coronary artery disease Eur Heart J 2005 26 10 967 974 10.1093/eurheartj/ehi190 15774493

[B10] Kjekshus JK Importance of heart rate in determining beta-blocker efficacy in acute and long-term acute myocardial infarction intervention trials Am J Cardiol 1986 57 12 43F 49F 10.1016/0002-9149(86)90888-X 2871745

[B11] Boissel JP Leizorovicz A Picolet H Peyrieux JC Secondary prevention after high-risk acute myocardial infarction with low-dose acebutolol Am J Cardiol 1990 66 3 251 260 10.1016/0002-9149(90)90831-K 2195860

[B12] Kolloch R Legler UF Champion A Cooper-Dehoff RM Handberg E Zhou Q Pepine CJ Impact of resting heart rate on outcomes in hypertensive patients with coronary artery disease: findings from the INternational VErapamil-SR/trandolapril STudy (INVEST) Eur Heart J 2008 29 10 1327 1334 18375982 10.1093/eurheartj/ehn123 PMC2805436

[B13] Williams B Lacy PS Impact of heart rate on central aortic pressures and hemodynamics: analysis from the CAFE (Conduit Artery Function Evaluation) study: CAFE-Heart Rate J Am Coll Cardiol 2009 54 8 705 713 10.1016/j.jacc.2009.02.088 19679248

[B14] Bangalore S Steg G Deedwania P Crowley K Eagle KA Goto S Ohman EM Cannon CP Smith SC Zeymer U beta-Blocker use and clinical outcomes in stable outpatients with and without coronary artery disease JAMA 2012 308 13 1340 1349 10.1001/jama.2012.12559 23032550

[B15] Fox K Borer JS Camm AJ Danchin N Ferrari R Lopez Sendon JL Steg PG Tardif JC Tavazzi L Tendera M Resting heart rate in cardiovascular disease J Am Coll Cardiol 2007 50 9 823 830 10.1016/j.jacc.2007.04.079 17719466

[B16] Verrier RL Tan A Heart rate, autonomic markers, and cardiac mortality Heart Rhythm 2009 6 11 Suppl S68 S75 19880076 10.1016/j.hrthm.2009.07.017 PMC2776094

[B17] Huikuri HV Jokinen V Syvänne M Nieminen MS Airaksinen KE Ikäheimo MJ Koistinen JM Kauma H Kesäniemi AY Majahalme S Heart rate variability and progression of coronary atherosclerosis Arterioscler Thromb Vasc Biol 1999 19 8 1979 1985 10.1161/01.ATV.19.8.1979 10446081

[B18] Rubin J Blaha MJ Budoff MJ Rivera JJ Shaw LJ Blankstein R Mallah MA Carr JJ Jones DL Blumenthal RS The relationship between resting heart rate and incidence and progression of coronary artery calcification: the Multi-Ethnic Study of Atherosclerosis (MESA) Atherosclerosis 2012 220 1 194 200 10.1016/j.atherosclerosis.2011.06.033 21763655

[B19] Lantelme P Mestre C Lievre M Gressard A Milon H Heart rate: an important confounder of pulse wave velocity assessment Hypertension 2002 39 6 1083 1087 10.1161/01.HYP.0000019132.41066.95 12052846

[B20] Albaladejo P Laurent P Pannier B Achimastos A Safar M Benetos A Influence of sex on the relation between heart rate and aortic stiffness J Hypertens 2003 21 3 555 562 10.1097/00004872-200303000-00021 12640249

[B21] Chen W Srinivasan SR Berenson GS Differential impact of heart rate on arterial wall stiffness and thickness in young adults: The Bogalusa Heart Study J Am Soc Hypertens 2008 2 3 152 157 10.1016/j.jash.2007.10.008 20409897

[B22] Park BJ Lee HR Shim JY Lee JH Jung DH Lee YJ Association between resting heart rate and arterial stiffness in Korean adults Arch Cardiovasc Dis 2010 103 4 246 252 10.1016/j.acvd.2010.03.004 20656635

[B23] O’Rourke MF Hashimoto J Mechanical factors in arterial aging: a clinical perspective J Am Coll Cardiol 2007 50 1 1 13 10.1016/j.jacc.2006.12.050 17601538

[B24] Wilkinson IB MacCallum H Flint L Cockcroft JR Newby DE Webb DJ The influence of heart rate on augmentation index and central arterial pressure in humans J Physiol 2000 525 Pt 1 263 270 10811742 10.1111/j.1469-7793.2000.t01-1-00263.x PMC2269933

[B25] Weber T Auer J O’Rourke MF Kvas E Lassnig E Berent R Eber B Arterial stiffness, wave reflections, and the risk of coronary artery disease Circulation 2004 109 2 184 189 10.1161/01.CIR.0000105767.94169.E3 14662706

[B26] Sugawara J Komine H Hayashi K Maeda S Matsuda M Relationship between augmentation index obtained from carotid and radial artery pressure waveforms J Hypertens 2007 25 2 375 381 10.1097/HJH.0b013e32801092ae 17211244

[B27] Black HR Greenberg BH Weber MA The foundation role of beta blockers across the cardiovascular disease spectrum: a year 2009 update Am J Med 2010 123 11 S2 10.1016/j.amjmed.2010.08.003 21035578

[B28] Rule AD Larson TS Bergstralh EJ Slezak JM Jacobsen SJ Cosio FG Using serum creatinine to estimate glomerular filtration rate: accuracy in good health and in chronic kidney disease Ann Intern Med 2004 141 12 929 937 10.7326/0003-4819-141-12-200412210-00009 15611490

[B29] Kööbi T Non-invasive cardiac output determination: state of the art Curr Opin Anaesthesiol 1999 12 1 9 13 10.1097/00001503-199902000-00003 17013291

[B30] Kööbi T Kähönen M Iivainen T Turjanmaa V Simultaneous non-invasive assessment of arterial stiffness and haemodynamics - a validation study Clin Physiol Funct Imaging 2003 23 1 31 36 10.1046/j.1475-097X.2003.00465.x 12558611

[B31] Kööbi T Kaukinen S Turjanmaa VM Uusitalo AJ Whole-body impedance cardiography in the measurement of cardiac output Crit Care Med 1997 25 5 779 785 10.1097/00003246-199705000-00012 9187596

[B32] Gorlin R Mc MI Medd WE Matthews MB Daley R Dynamics of the circulation in aortic valvular disease Am J Med 1955 18 6 855 870 10.1016/0002-9343(55)90169-8 14376408

[B33] British-Standards-Institution Precicion of test methods 1: Guide for the determination and reproducability for a standard test method (BS 5497) 1979 London: British Standards Institution

[B34] Tahvanainen A Koskela J Tikkakoski A Lahtela J Leskinen M Kähönen M Nieminen T Kööbi T Mustonen J Pörsti I Analysis of cardiovascular responses to passive head-up tilt using continuous pulse wave analysis and impedance cardiography Scand J Clin Lab Invest 2009 69 1 128 137 10.1080/00365510802439098 18850486

[B35] Tahvanainen AM Tikkakoski AJ Leskinen MH Nordhausen K Kahonen M Koobi T Mustonen JT Porsti IH Supine and upright haemodynamic effects of sublingual nitroglycerin and inhaled salbutamol: a double-blind, placebo-controlled, randomized study J Hypertens 2012 30 2 297 306 10.1097/HJH.0b013e32834e4b26 22179079

[B36] Chen CH Nevo E Fetics B Pak PH Yin FC Maughan WL Kass DA Estimation of central aortic pressure waveform by mathematical transformation of radial tonometry pressure. Validation of generalized transfer function Circulation 1997 95 7 1827 1836 10.1161/01.CIR.95.7.1827 9107170

[B37] Avolio A Parati G Reflecting on posture J Hypertens 2011 29 4 655 657 10.1097/HJH.0b013e328345852a 21389813

[B38] Tikkakoski AJ Tahvanainen AM Leskinen MH Koskela JK Haring A Viitala J Kähönen MA Kööbi T Niemelä O Mustonen JT Hemodynamic alterations in hypertensive patients at rest and during passive head-up tilt J Hypertens 2013 31 5 906 915 10.1097/HJH.0b013e32835ed605 23412427

[B39] Palatini P Julius S Heart rate and the cardiovascular risk J Hypertens 1997 15 1 3 17 10.1097/00004872-199715010-00001 9050965

[B40] Magder SA The ups and downs of heart rate Crit Care Med 2012 40 1 239 245 10.1097/CCM.0b013e318232e50c 22179340

[B41] Tanaka N Nozawa T Yasumura Y Futaki S Hiramori K Suga H Heart-rate-proportional oxygen consumption for constant cardiac work in dog heart Jpn J Physiol 1990 40 4 503 521 10.2170/jjphysiol.40.503 2077175

[B42] Palatini P Julius S Elevated heart rate: a major risk factor for cardiovascular disease Clin Exp Hypertens 2004 26 7–8 637 644 15702618 10.1081/ceh-200031959

[B43] Palatini P Role of elevated heart rate in the development of cardiovascular disease in hypertension Hypertension 2011 58 5 745 750 10.1161/HYPERTENSIONAHA.111.173104 21896939

[B44] Grassi G Sympathetic neural activity in hypertension and related diseases Am J Hypertens 2010 23 10 1052 1060 10.1038/ajh.2010.154 20651696

[B45] Julius S Esler MD Randall OS Role of the autonomic nervous system in mild human hypertension Clin Sci Mol Med Suppl 1975 2 243s 252s 802640 10.1042/cs048243s

[B46] Wilkinson IB Mohammad NH Tyrrell S Hall IR Webb DJ Paul VE Levy T Cockcroft JR Heart rate dependency of pulse pressure amplification and arterial stiffness Am J Hypertens 2002 15 1 Pt 1 24 30 11824855 10.1016/s0895-7061(01)02252-x

[B47] Millasseau SC Stewart AD Patel SJ Redwood SR Chowienczyk PJ Evaluation of carotid-femoral pulse wave velocity: influence of timing algorithm and heart rate Hypertension 2005 45 2 222 226 10.1161/01.HYP.0000154229.97341.d2 15642772

[B48] Sa Cunha R Pannier B Benetos A Siche JP London GM Mallion JM Safar ME Association between high heart rate and high arterial rigidity in normotensive and hypertensive subjects J Hypertens 1997 15 12 Pt 1 1423 1430 9431848 10.1097/00004872-199715120-00009

[B49] Nurnberger J Opazo Saez A Dammer S Mitchell A Wenzel RR Philipp T Schafers RF Left ventricular ejection time: a potential determinant of pulse wave velocity in young, healthy males J Hypertens 2003 21 11 2125 2132 10.1097/00004872-200311000-00022 14597856

[B50] Dart AM Kingwell BA Pulse pressure–a review of mechanisms and clinical relevance J Am Coll Cardiol 2001 37 4 975 984 10.1016/S0735-1097(01)01108-1 11263624

[B51] Plichart M Thomas F Empana JP Bean K Perier MC Celermajer DS Hanon O Danchin N Pannier B Jouven X Gender-specific trends in heart rate in the general population from 1992–2007: a study of 226,288 French adults Eur J Prev Cardiolog 2013 20 1 61 72 10.1177/2047487311434231 22345675

[B52] Peltonen M Harald K Männistö S Saarikoski L Peltomäki P Lund L Sundvall J Juolevi A Laatikainen T Aldén-Nieminen H Kansallinen Finriski 2007 -terveystutkimus Tutkimuksen toteutus ja tulokset. Kansanterveyslaitoksen julkaisuja B 34/2008 2008 Yliopistopaino: Helsinki

[B53] Sharman JE Lim R Qasem AM Coombes JS Burgess MI Franco J Garrahy P Wilkinson IB Marwick TH Validation of a generalized transfer function to noninvasively derive central blood pressure during exercise Hypertension 2006 47 6 1203 1208 10.1161/01.HYP.0000223013.60612.72 16651459

[B54] Buckberg GD Fixler DE Archie JP Hoffman JI Experimental subendocardial ischemia in dogs with normal coronary arteries Circ Res 1972 30 1 67 81 10.1161/01.RES.30.1.67 5007529

