# Supplementary material for: Association of resting heart rate with cardiovascular function: a cross-sectional study in 522 Finnish subjects
Source: BMC Cardiovasc Disord. 2013 Nov 15;13:102. doi: 10.1186/1471-2261-13-102 (PMC3832902; doi:10.1186/1471-2261-13-102)
Supplement: Additional file 1 — Regular medications used by the study population. [file 1471-2261-13-102-S1.docx]

# **Additional file 1**

# Association of resting heart rate with cardiovascular function: a cross-sectional study in 522 Finnish subjects

Jenni K. KOSKELA^1^, Anna TAHVANAINEN^1^, Antti HARING^2^, Antti J. TIKKAKOSKI^1^, Erkki ILVESKOSKI^3^, Jani VIITALA^1^, Miia H. LESKINEN^1^, Terho LEHTIMÄKI^4,5^, Mika A.P. KÄHÖNEN^6,7^, Tiit KÖÖBI^7^, Onni NIEMELÄ^8^, Jukka T. MUSTONEN^1,9^ and Ilkka H. PÖRSTI^1,9^

**Additional file 1** Regular medications used by the study population.

|  | Resting heart rate tertiles | | |  |
| --- | --- | --- | --- | --- |
|  | 1 | 2 | 3 | All |
|  | n=172 | n=176 | n=174 | n=522 |
| Allopurinol | 1 | 0 | 1 | 2 |
| Androgens | 1 | 0 | 0 | 1 |
| Antidepressants | 14 | 5 | 10 | 29 |
| Antiepileptic agents | 2 | 1 | 2 | 5 |
| Antihistamine | 4 | 5 | 7 | 16 |
| Anxiolytic agents | 2 | 0 | 1 | 3 |
| Bisphosphonate | 1 | 0 | 0 | 1 |
| Carbimazole | 1 | 0 | 0 | 1 |
| Corticosteroid, intranasal or inhaled | 6 | 4 | 5 | 15 |
| Dopamine agonists | 0 | 0 | 1 | 1 |
| Female hormones* | 26 | 30 | 26 | 82 |
| Glucosamine | 2 | 4 | 0 | 6 |
| Hypolipidemic agents (statins) | 4 | 3 | 6 | 13 |
| Isotretinoin | 0 | 1 | 0 | 1 |
| Melatonin | 1 | 0 | 1 | 2 |
| NSAID | 2 | 1 | 1 | 4 |
| Oxybutynin | 0 | 0 | 1 | 1 |
| Proton pump inhibitors^†^ | 0 | 5 | 9 | 14 |
| Salazosulphapyridine | 0 | 1 | 0 | 1 |
| Tamoxifen | 0 | 1 | 0 | 1 |
| Tamsulosin | 1 | 1 | 0 | 2 |
| Thyroxin | 4 | 5 | 6 | 15 |
| Warfarin | 0 | 1 | 0 | 1 |

NSAID, non-steroidal anti-inflammatory agents; *low-dose oestrogen, progestin, and combination products (intrauterine or per oral); ^†^the use of proton pump inhibitors was not equally distributed between the tertiles (p=0.012, χ^2^ test), but mean heart rate did not differ between subjects medicated and not-medicated with proton pump inhibitors (p>0.050, T-test).
